# The potential risk factors of postoperative cognitive dysfunction for endovascular therapy in acute ischemic stroke with general anesthesia

**DOI:** 10.1515/med-2024-1085

**Published:** 2024-12-17

**Authors:** Yangning Zhou, Yan Wang, Limin Xu

**Affiliations:** Department of Anesthesiology, Shanghai United Family Hospital, Shanghai 200050, China; Department of Anesthesiology, Shanghai SinoUnited Hospital, No. 350 Middle Jiangxi Road, Shanghai 200001, China

**Keywords:** cognitive impairment, acute ischemic stroke, nomogram, prediction, anesthesia depth

## Abstract

**Background:**

Postoperative cognitive dysfunction (POCD) frequently occurs following endovascular therapy for acute ischemic stroke (AIS). Given the complexity of predicting AIS clinically, there is a pressing need to develop a preemptive prediction model and investigate the impact of anesthesia depth on AIS.

**Methods:**

A total of 333 patients diagnosed with AIS were included in the study, comprising individuals with non-POCD (*n* = 232) or POCD (*n* = 101). Univariate and multivariate logistic regression analyses were utilized to examine the independent risk factors associated with POCD. A calibration, decision curve analysis, and precision–recall curves were employed to assess the model’s goodness of fit.

**Results:**

Multivariate regression analysis identified two inflammatory indicators, high-sensitivity C reactive protein (hs-CRP) and systemic immune inflammatory index (SII), and three brain injury indicators, National Institute of Health Stroke Scale (NIHSS) score, N-terminal pro-brain natriuretic peptide (NT-proBNP), and soluble protein-100 β (S100-β), which were used to construct a nomogram model.

**Conclusion:**

The composite predictive model incorporating NIHSS score, hs-CRP, SII, NT-proBNP, and S100-β demonstrated efficacy in predicting POCD following AIS. Additionally, our results suggest a potential association between depth of anesthesia, cognitive impairment, and inflammatory response in AIS patients.

## 1 Introduction

Postoperative cognitive dysfunction (POCD) is characterized by impairments in memory, abstract thinking, and orientation following anesthesia surgery, often accompanied by a decrease in social activity. This includes changes in personality, social ability, cognitive function, and skills [1,2]. More than 20% of surgical patients may experience postoperative delirium on the first day following surgery, and between 10 and 25% may develop POCD within 3–6 months postoperatively [3]. The prevalence of POCD is particularly high among elderly patients post-surgery, and a study reported that the incidence of POCD in elderly patients following major noncardiac surgery was 25.8% [4]. The pathogenesis of POCD may involve neuroinflammation, mitochondrial dysfunction, oxidative stress, blood–brain barrier disruption, and damage to the brain–gut axis [2,5–7]; however, the etiology of POCD remains incompletely elucidated. Empirical evidence from clinical investigations suggests that variables including anesthesia modality, surgical duration, patient age, educational attainment, American Society of Anesthesiologists classification, surgical type, length of hospitalization, incidence of delirium, and opioid utilization may serve as predictive indicators of cognitive decline following surgery [1,8–11]. Consequently, a thorough examination of the risk factors associated with POCD can offer valuable insights into its underlying pathophysiological mechanisms.

Acute ischemic stroke (AIS) is the most prevalent form of stroke, representing approximately 70% of all cases [12,13]. The timely restoration of blocked vascular perfusion, preservation of ischemic penumbra, reduction of core infarction volume, and prompt vascular recanalization are crucial for the effective treatment of AIS patients and can significantly enhance patient prognosis [12,13]. Furthermore, there is a growing interest in the impact of anesthesia techniques and management on neurological outcomes following endovascular therapy (EVT) in individuals with AIS [14,15]. The anesthesia methods most frequently utilized for EVT in AIS patients are local anesthesia/conscious sedation and general anesthesia, each presenting distinct advantages and disadvantages and varying impacts on neurological outcomes. Local/conscious sedation offers the benefit of allowing the patient to remain conscious throughout the procedure, facilitating the assessment of neurological function [14,15]. The initiation of surgical treatment demonstrates a brief start-up time, with perioperative hemodynamics remaining stable. However, notable drawbacks include patient agitation and uncooperativeness, intraoperative body movements leading to treatment time prolongation, potential risks of respiratory depression, carbon dioxide retention, and reflux aspiration in the absence of airway protection [14,15]. General anesthesia offers the advantage of airway control and protection against reflux aspiration, facilitating ease of patient management and procedural execution. While artificial airway placement aids in ventilation control, it also presents significant disadvantage, such as pneumonia [14–16]. Studies have shown conflicting results regarding the impact of anesthesia type on the neurological prognosis of patients with surgical operation [17,18]. No statistically significant variance in the prevalence of POCD was observed between general anesthesia and regional anesthesia in elderly patients undergoing hip fracture surgery. Comparable outcomes were noted for both methods at 24 h, 3 days, and 7 days postoperatively [10,19–21]. Conscious sedation does not demonstrate superior efficacy compared to general anesthesia in EVT for patients with post-circulatory AIS, particularly with respect to the primary outcome of functional recovery, and may even be inferior in terms of the secondary outcome of successful reperfusion [22,23]. Furthermore, research has demonstrated that among ischemic stroke patients undergoing EVT, general anesthesia is associated with a higher rate of recurrence and enhanced functional recovery at the 3-month mark when compared to non-general anesthesia techniques [24]. General anesthesia is advised to be prioritized as the preferred option in the majority of EVT interventions for AIS [24].

These studies indicate that general anesthesia may offer advantages in the prognosis of EVT for AIS. Nevertheless, there remains uncertainty surrounding the selection of risk factors linked to perioperative cognitive impairment and prognostic markers associated with general anesthesia. In this study, we collected general clinical data, markers of brain injury, and inflammatory markers from patients undergoing EVT under general anesthesia for AIS patients in order to identify independent prognostic risk factors associated with POCD and establish the relevant prediction model.

## 2 Methods

### 2.1 Study participants and design

**Figure 1:**
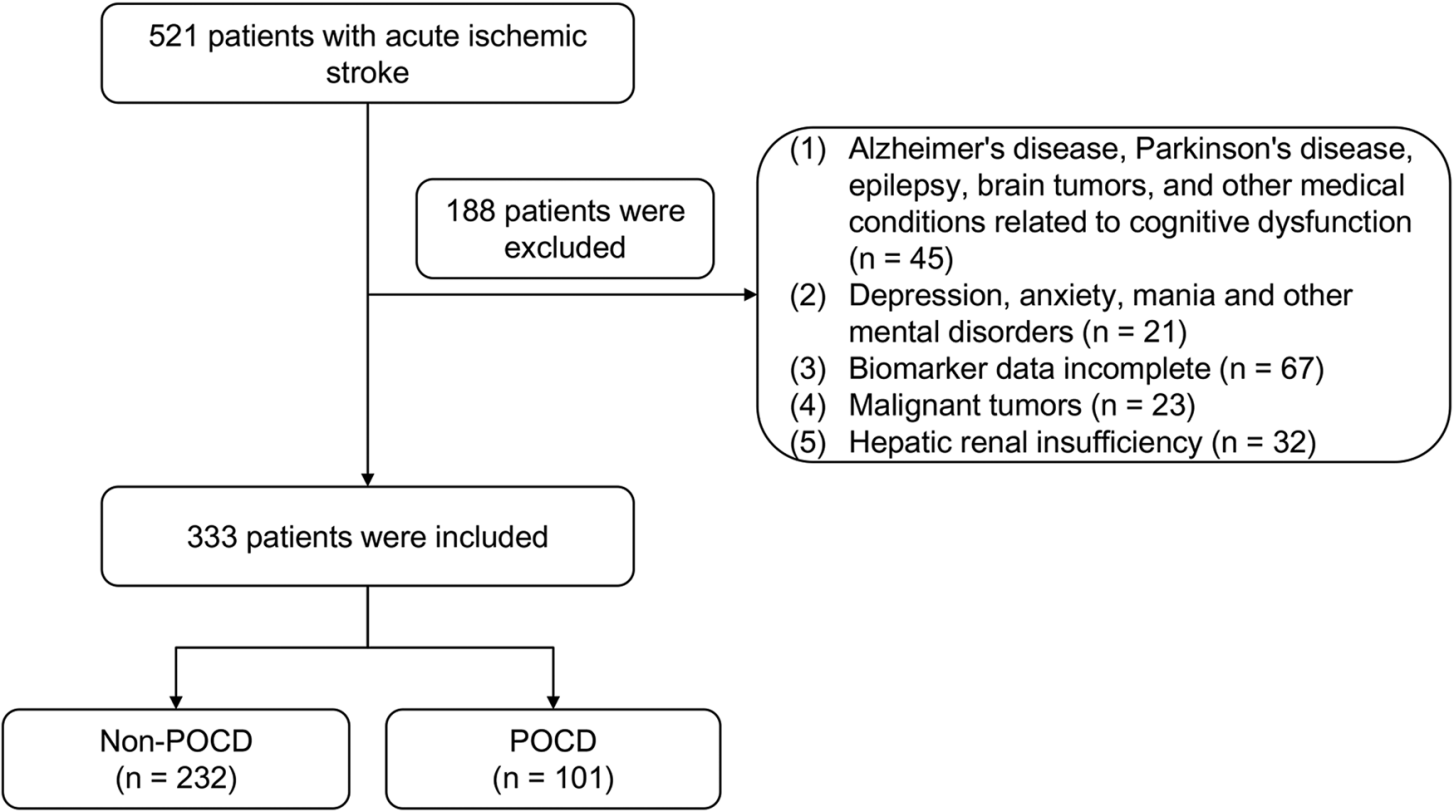
A flowchart about the criteria for exclusion: 521 patients with AIS were recruited and 188 patients were excluded. Ultimately, a total of 333 patients were included in the study.

A retrospective study was conducted to collect clinical data from 521 patients with AIS between January 2019 and January 2024. Inclusion criteria for the study required a diagnosis of AIS based on the guideline of the American Heart Association/American Stroke Association [25]. The study excluded participants based on the following criteria: (1) Alzheimer’s disease, Parkinson’s disease, epilepsy, brain tumors, and other medical conditions related to cognitive dysfunction; (2) depression, anxiety, mania, and other mental disorders; (3) biomarker data incomplete; (4) malignant tumors; and (5) hepatic renal insufficiency. About 188 AIS patients were excluded. Ultimately, a total of 333 patients diagnosed with AIS were included in the study, comprising individuals with non-POCD (*n* = 232) or POCD (*n* = 101) as shown in Figure 1. The authors bear responsibility for all facets of the research, including addressing any concerns regarding the accuracy or integrity of the work.

### 2.2 Data collection

General information, including age, gender, body mass index (BMI), time of operation, Trial of ORG 10172 in acute stroke treatment (TOAST) classification, level of education, smoking history, diseases history (hypertension history and diabetes history), length of stay (LOS), systolic blood pressure (SBP), diastolic blood pressure (DBP), National Institute of Health Stroke Scale (NIHSS), bispectral index (BIS), biochemical markers, such as creatinine (Cre), homocysteine (Hcy), uric acid (UA), d-dimer (d-D), high-sensitivity C reactive protein (hs-CRP), platelet lymphocyte ratio (PLR), neutrophil lymphocyte ratio (NLR), systemic inflammatory response index (SIRI), systemic immune inflammatory index (SII), triglyceride (TG), total cholesterol (TC), low density lipoprotein cholesterol (LDL-C), high-density lipoprotein cholesterol (HDL-C), N-terminal pro-brain natriuretic peptide (NT-proBNP), and soluble protein-100 β (S100-β) were collected to filtrate POCD related risk factors. POCD is defined as a Mini-Mental State Examination (MMSE) score of 26 points or lower, while non-POCD is operationally defined as an MMSE score ranging from 27 to 30. In addition, a total of 227 patients diagnosed with AIS were included, comprising individuals with high BIS (*n* = 163) or low BIS (*n* = 64) to analyze the correlation of BIS with clinical parameters in AIS patients. The low BIS group is defined as individuals scoring between 30 and 49, while the high BIS group is characterized by scores falling between 50 and 60.

### 2.3 Statistical methods

Statistical analyses were performed using IBM SPSS Statistics (version 25.0, IBM, Armonk, NY, USA), GraphPad Prism 9.0 (GraphPad Software, Inc., La Jolla, CA, USA), R software (version 4.2.1) with the rms package (version 6.3-0), pROC package (version 1.18.0), and ggplot2 package (version 3.3.6). Descriptive statistics, such as the median and interquartile range for non-normally distributed data or the mean and standard deviation for normally distributed data, were calculated. The study utilized Fisher’s exact test or chi-square test for categorical variables, Wilcoxon signed-rank test for continuous variables, *t*-tests, and Mann–Whitney test for group differences assessment. The independent risk factors associated with POCD were examined through binary logistic regression analysis. Receiver operating characteristic (ROC) curves with the area under the curve (AUC) was used to evaluate the diagnostic performance, in addition to indicators including sensitivity, specificity, positive predictive value (PPV), negative predictive value (NPV), and cut-off value, which were computed using optimal thresholds derived from ROC curve analysis. The glm function was utilized to develop a binary logistic model, while the rms (version 6.4.0) and ResourceSelection (version 0.3-5) packages were employed to construct a nomogram model and facilitate its visualization. The goodness-of-fit of the nomogram model was assessed using statistical measures such as the likelihood-ratio test, C index, and Hosmer-Lemeshow goodness fit. The stdca.R file was employed for conducting decision curve analysis (DCA). The rms package (version 6.3-0) was utilized for calibration analysis and data visualization. The pROC package (version 1.18.0) was utilized to conduct precision–recall (PR) analysis on the dataset, and the outcomes were graphically represented using ggplot2 (version 3.3.6). A two-tailed approach was employed for all statistical analyses, with statistical significance defined as *P*-values less than 0.05.


**Ethical approval:** The study adhered to the Declaration of Helsinki (2013 revision) and received approval from the Ethics Committees of the Shanghai United Family Hospital and Shanghai SinoUnited Hospital (Approval number: 20240089).

## 3 Results

### 3.1 Baseline subject’s characteristics and risk factors of POCD

A total of 333 patients diagnosed with AIS were included in the study, comprising individuals with non-POCD (*n* = 232) or POCD (*n* = 101). Baseline data analysis revealed that several key indicators, including DBP, atrial fibrillation incidents, NIHSS score, d-D, hs-CRP, NLR, SIRI, SII, NT-proBNP, as well as the S100-β, were significantly elevated in the POCD group compared to the non-POCD group (Table 1). In order to investigate independent risk factors linked to POCD, we employed univariate and multivariate logistic regression analyses on multiple clinical parameters. Our findings revealed that NIHSS score, hs-CRP, SII, NT-proBNP, and S100-β were identified as independent risk factors for POCD (Table 2).

**Table 1:** Baseline characteristics of the study population

Characteristics	Non-POCD (*N* = 232)	POCD (*N* = 101)	*P* value	Statistical method
Age (years), median (IQR)	64 (58, 71)	63 (55, 72)	0.491	Wilcoxon
Gender, *n* (%)			0.847	Chisq test
Male	156 (46.8%)	69 (20.7%)		
Female	76 (22.8%)	32 (9.6%)		
Time of operation (min), median (IQR)	84 (72, 103)	90 (75, 102)	0.280	Wilcoxon
TOAST classification, *n* (%)			0.293	Chisq test
Small vessel occlusion	89 (26.7%)	28 (8.4%)		
Cardioembolism	47 (14.1%)	24 (7.2%)		
Large-artery atherosclerosis	72 (21.6%)	35 (10.5%)		
Others	24 (7.2%)	14 (4.2%)		
TICI score, *n* (%)			0.687	Chisq test
2b-3	208 (62.5%)	92 (27.6%)		
0-2a	24 (7.2%)	9 (2.7%)		
Pneumonia, *n* (%)			0.226	Chisq test
Yes	92 (27.6%)	33 (9.9%)		
No	140 (42.0%)	68 (20.4%)		
Level of education, *n* (%)			0.470	Chisq test
High school or higher	80 (24.0%)	39 (11.7%)		
Junior middle school or lower	152 (45.6%)	62 (18.6%)		
LOS (days), median (IQR)	10 (8, 12)	9 (7, 11)	0.957	Wilcoxon
BMI (kg/m^2^), median (IQR)	25.4 (22.3, 26.6)	25.8 (23.7, 26.6)	0.180	Wilcoxon
Hypertension history, *n* (%)			0.705	Chisq test
No	152 (45.6%)	64 (19.2%)		
Yes	80 (24.0%)	37 (11.1%)		
SBP (mmHg), median (IQR)	150 (137, 161)	151 (134, 157)	0.078	Wilcoxon
DBP (mmHg), median (IQR)	87 (80, 96.25)	93 (83, 97)	0.024	Wilcoxon
Atrial fibrillation, *n* (%)			0.027	Chisq test
No	158 (47.4%)	56 (16.8%)		
Yes	74 (22.2%)	45 (13.5%)		
Diabetes history, *n* (%)			0.940	Chisq test
Yes	86 (25.8%)	37 (11.1%)		
No	146 (43.8%)	64 (19.2%)		
Stroke history, *n* (%)			0.555	Chisq test
No	157 (47.1%)	65 (19.5%)		
Yes	75 (22.5%)	36 (10.8%)		
NIHSS score, median (IQR)	4 (3, 5)	8 (7, 9)	0.000	Wilcoxon
Hemoglobin (g/dL), median (IQR)	11.2 (10.9, 11.7)	11.0 (10.8, 11.3)	0.227	Wilcoxon
Cre (mg/dL), median (IQR)	1.1 (0.9, 1.5)	1.2 (1.0, 1.5)	0.162	Wilcoxon
Hcy (μmol/L), median (IQR)	16.95 (14.6, 21.1)	18 (16.0, 21.1)	0.397	Wilcoxon
UA (μmol/L), median (IQR)	310.9 (179.7, 414.7)	277.1 (175.9, 399.2)	0.632	Wilcoxon
d-D (mg/L), median (IQR)	1.0 (0.8, 1.7)	1.6 (1.4, 1.7)	0.000	Wilcoxon
hs-CRP (mg/L), median (IQR)	4.6 (3.8, 5.4)	8.7 (7.7, 8.9)	0.000	Wilcoxon
PLR, median (IQR)	125.6 (114.6, 140.4)	133.7 (118.9, 142.2)	0.083	Wilcoxon
NLR, median (IQR)	2.7 (2.4, 3.1)	4.1 (3.8, 4.3)	0.000	Wilcoxon
SIRI, median (IQR)	1.6 (1.3, 2.3)	3.4 (3.1, 4.1)	0.000	Wilcoxon
SII, median (IQR)	460.9 (411.2, 562.1)	701.3 (583.4, 804.6)	0.000	Wilcoxon
TG (mmol/L), median (IQR)	2.0 (1.8, 2.1)	2.0 (1.8, 2.1)	0.248	Wilcoxon
TC (mmol/L), median (IQR)	4.3 (3.9, 5.1)	4.2 (3.9, 4.7)	0.046	Wilcoxon
LDL-C (mmol/L), median (IQR)	3.1 (2.7, 3.4)	3 (2.8, 3.3)	0.472	Wilcoxon
HDL-C (mmol/L), median (IQR)	1.2 (1.0, 1.4)	1.2 (1.0, 1.4)	0.594	Wilcoxon
NT-proBNP (ng/L), median (IQR)	406.6 (364.4, 477.6)	563.2 (425.5, 654.4)	0.000	Wilcoxon
S100-β (ng/L), median (IQR)	139.9 (126.4, 159.9)	232.2 (212.5, 262.2)	0.000	Wilcoxon

POCD, postoperative cognitive dysfunction; IQR, interquartile range; TOAST, Trial of ORG 10172 in acute stroke treatment; LOS, length of stay; BMI, body mass index; SBP, systolic blood pressure; DBP, diastolic blood pressure; NIHSS, National Institute of Health Stroke Scale; TICI, thrombolysis in cerebral infarction; Cre, creatinine; Hcy, homocysteine; UA, uric acid; d-D, d-dimer; hs-CRP, high-sensitivity C reactive protein; PLR, platelet lymphocyte ratio; NLR, neutrophil lymphocyte ratio; SIRI, systemic inflammatory response index; SII, systemic immune inflammatory index; TG, triglyceride; TC, total cholesterol; LDL-C, low density lipoprotein cholesterol; HDL-C, high-density lipoprotein cholesterol; NT-proBNP, N-terminal pro-brain natriuretic peptide; S100-β, soluble protein-100 β.

**Table 2:** Univariate and multivariate analysis of POCD related risk factors in AIS patients

Characteristics	Total (*N*)	OR (95% CI) Univariate analysis	*P* value	OR (95% CI) Multivariate analysis	*P* value
Age (years)	333	0.99 (0.96–1.02)	0.484		
Gender	333				
Male	225	Reference			
Female	108	0.95 (0.58–1.57)	0.847		
Time of operation (min)	333	1.01 (0.99–1.02)	0.330		
Pneumonia	333				
Yes	125	Reference			
No	208	1.35 (0.83–2.22)	0.227		
Level of education	333				
High school or higher	119	Reference			
Junior middle school or lower	214	0.84 (0.52–1.36)	0.470		
LOS (days)	333	1.01 (0.91–1.11)	0.924		
BMI (kg/m^2^)	333	1.08 (0.98–1.19)	0.131		
Hypertension history	333				
No	216	Reference			
Yes	117	1.09 (0.68–1.79)	0.706		
SBP (mmHg)	333	0.98 (0.97–1.00)	0.044	1.01 (0.89–1.14)	0.817
DBP (mmHg)	333	1.03 (1.01–1.06)	0.025	0.96 (0.79–1.17)	0.704
Atrial fibrillation	333				
No	214	Reference		Reference	
Yes	119	1.72 (1.06–2.77)	0.027	1.49 (0.07–32.20)	0.798
Stroke history	333				
No	222	Reference			
Yes	111	1.16 (0.71–1.89)	0.555		
NIHSS score	333	4.18 (3.01–5.79)	<0.001	15.59 (2.14–113.49)	0.007
Hemoglobin (g/dL)	333	1.16 (0.94–1.42)	0.164		
Cre (mg/dL)	333	1.56 (0.82–2.94)	0.168		
Hcy (μmol/L)	333	1.04 (0.97–1.11)	0.295		
UA (μmol/L)	333	1.00 (0.99–1.00)	0.620		
d-D (mg/L)	333	14.28 (7.03–28.97)	<0.001	0.38 (0.01–21.18)	0.637
hs-CRP (mg/L)	333	2.73 (2.24–3.34)	<0.001	3.42 (1.34–8.72)	0.010
PLR	333	1.02 (0.99–1.03)	0.071	1.09 (0.98–1.23)	0.113
SII	333	1.01 (1.01–1.02)	<0.001	1.04 (1.00–1.07)	0.034
TG (mmol/L)	333	1.60 (0.62–4.15)	0.331		
TC (mmol/L)	333	0.67 (0.47–0.96)	0.029	2.92 (0.25–34.49)	0.395
LDL-C (mmol/L)	333	0.87 (0.52–1.46)	0.599		
HDL-C (mmol/L)	333	1.34 (0.45–3.93)	0.598		
NT-proBNP (ng/L)	333	1.01 (1.01–1.01)	<0.001	1.01 (1.00–1.03)	0.039
S100-β (ng/L)	333	1.03 (1.03–1.04)	<0.001	1.07 (1.02–1.13)	0.007

POCD, postoperative cognitive dysfunction; AIS, acute ischemic stroke; OR, odds ratio; LOS, length of stay; BMI, body mass index; SBP, systolic blood pressure; DBP, diastolic blood pressure; NIHSS, National Institute of Health Stroke Scale; Cre, creatinine; Hcy, homocysteine; UA, uric acid; d-D, d-dimer; hs-CRP, high-sensitivity C reactive protein; PLR, platelet lymphocyte ratio; SII, systemic immune inflammatory index; TG, triglyceride; TC, serum total cholesterol; LDL-C, low density lipoprotein cholesterol; HDL-C, high-density lipoprotein cholesterol; NT-proBNP, N-terminal pro-brain natriuretic peptide; S100-β, soluble protein-100 β.

### 3.2 Diagnostic performance of risk factors for POCD in AIS patients

**Figure 2:**
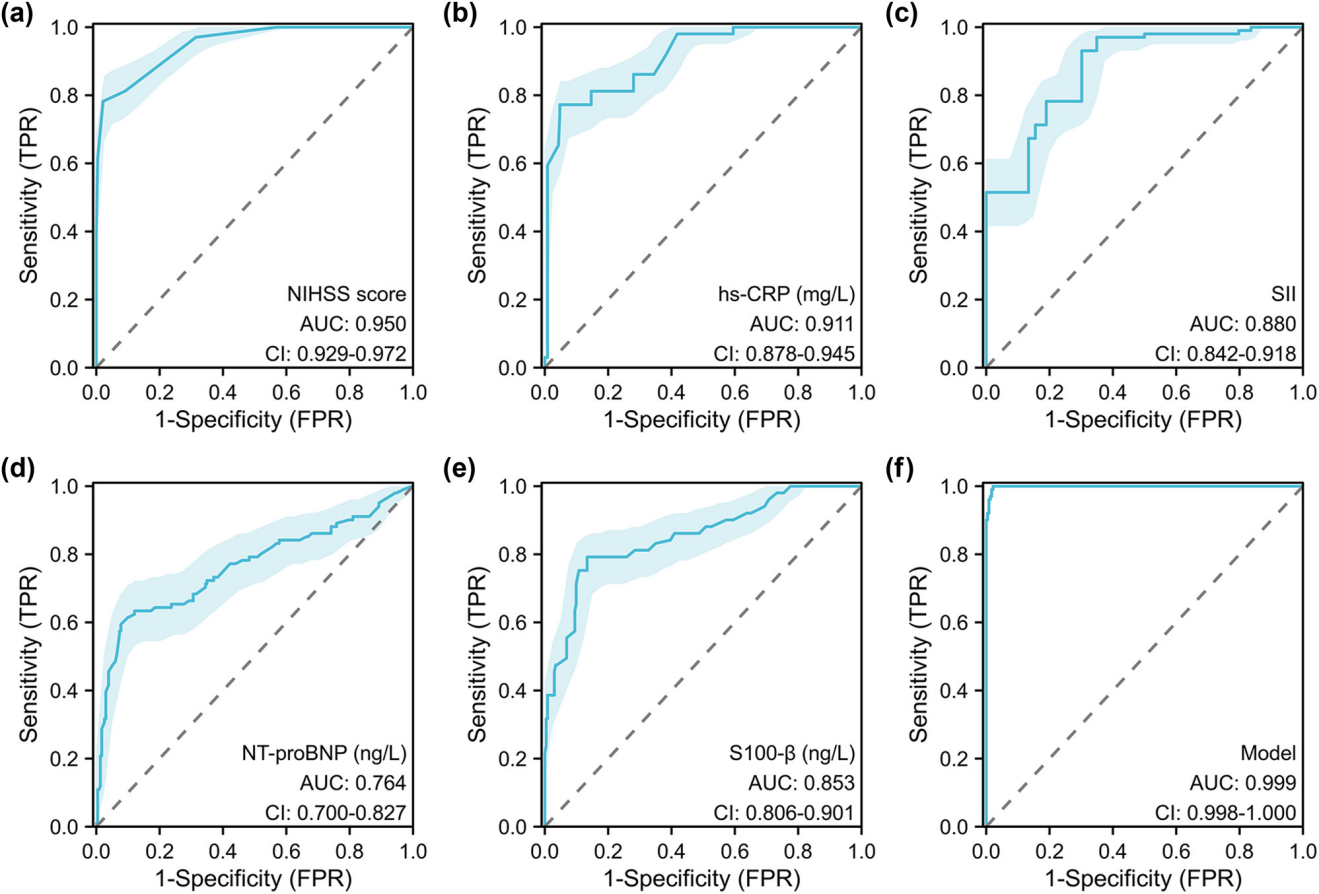
Diagnostic performance of risk factors for POCD in AIS patients. In order to investigate the diagnostic performance of NIHSS score (a), hs-CRP (b), SII (c), NT-proBNP (d), S100-β (e), and combination diagnosis model (f) in distinguishing between patients with POCD or non-POCD. ROC with AUC was used to evaluate the diagnostic performance in 333 AIS patients.

In order to investigate the diagnostic performance of NIHSS score, hs-CRP, SII, NT-proBNP, and S100-β in distinguishing between patients with POCD or non-POCD, a total of 333 AIS patients were recruited. The corresponding AUC values for NIHSS score (Figure 2a), hs-CRP (Figure 2b), SII (Figure 2c), NT-proBNP (Figure 2d), and S100-β (Figure 2e) were determined to be 0.950 (95% CI: 0.929–0.972), 0.911 (95% CI: 0.878–0.945), 0.880 (95% CI: 0.842–0.918), 0.764 (0.700–0.827), and 0.853 (0.806–0.901), respectively. The combination of five independent risk factors has excellent diagnostic efficacy for POCD (AUC = 0.999; 95% CI: 0.998–1.000) (Figure 2f). The cut-off value, sensitivity, specificity, accuracy, PPV, and NPV of NIHSS score, hs-CRP, SII, NT-proBNP, and S100-β for the diagnosis of POCD are shown in Table 3.

**Table 3:** Diagnostic performance of variables for POCD in AIS patients

Variables	Cut-off	Sensitivity	Specificity	Accuracy	PPV	NPV
NIHSS score	6.50	0.782	0.978	0.918	0.940	0.912
hs-CRP (mg/L)	7.40	0.772	0.953	0.898	0.876	0.9066
SII	521.800	0.931	0.698	0.769	0.573	0.959
NT-proBNP (ng/L)	520.200	0.594	0.922	0.822	0.769	0.839
S100-β (ng/L)	186.600	0.792	0.866	0.844	0.721	0.905

POCD, postoperative cognitive dysfunction; AIS, acute ischemic stroke; NIHSS, National Institute of Health Stroke Scale; hs-CRP, high-sensitivity C reactive protein; SII, systemic immune inflammatory index; NT-proBNP, N-terminal pro-brain natriuretic peptide; S100-β, soluble protein-100 β; PPV, positive predictive value; NPV, negative predictive value.

### 3.3 Develop a nomogram model for the evaluation of POCD risk

**Figure 3:**
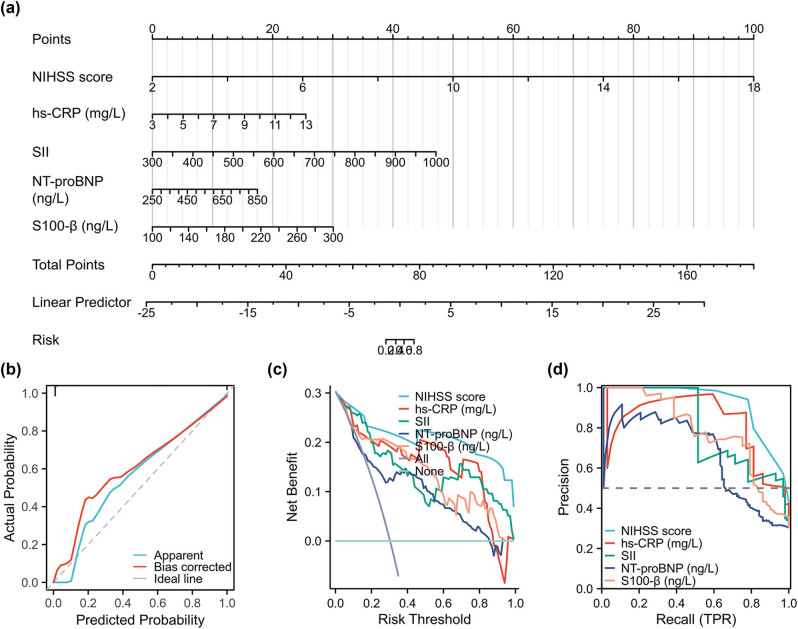
Develop a nomogram model for the evaluation of POCD risk. The independent risk factors for predicting POCD patients from AIS patients were determined using multiple logistic regression analysis. The nomogram effectively integrates these five predictors, NIHSS score, hs-CRP, SII, NT-proBNP, and S100-β (a). A calibration curve (b), DCA (c), and PR (d) curves were employed to assess the model’s goodness of fit.

The independent risk factors for predicting POCD patients from AIS patients were determined using multiple logistic regression analysis. The nomogram effectively integrates these five predictors, NIHSS score, hs-CRP, SII, NT-proBNP, and S100-β, and illustrates their interconnectedness within the predictive framework by means of calibrated line segments on a shared plane. The cumulative scores for the five variables were calculated, and subsequently used to determine the probability of POCD risk (Figure 3a). Calibration curves (Figure 3b), DCA (Figure 3c), and PR curves (Figure 3d) were used to evaluate the stability of nomogram models. The statistical analysis, including the likelihood-ratio test (*χ* ^2^ = 381.02; *P* < 0.001), C index (0.999; 95% CI: 0.998–1.000; *P* < 0.001), and Hosmer-Lemeshow goodness fit (*χ* ^2^ = 0.311; *P* = 1.000), indicates that the model exhibits a favorable level of fit (Figure 3b). DCA curve analysis is employed to depict the alteration in the net return value, considering the intervention condition and the predicted value of the model, as the risk probability threshold undergoes modification. The five variables surpass the reference line within a substantial interval of probability threshold, thereby signifying the efficacy of the variable model (Figure 3c). The PR graph is a curve that reflects the relationship between precision and recall. The AUC value of NIHSS score, hs-CRP, SII, NT-proBNP, and S100-β was 0.950 (95% CI: 0.929–0.972), 0.911 (95% CI: 0.878–0.945), 0.880 (95% CI: 0.842–0.918), 0.764 (95% CI: 0.700–0.827), and 0.853 (95% CI: 0.806–0.901), respectively, suggesting that five variables have good diagnostic effect in predicting POCD (Figure 3d).

### 3.4 Correlation of BIS with clinical parameters in AIS patients

**Figure 4:**
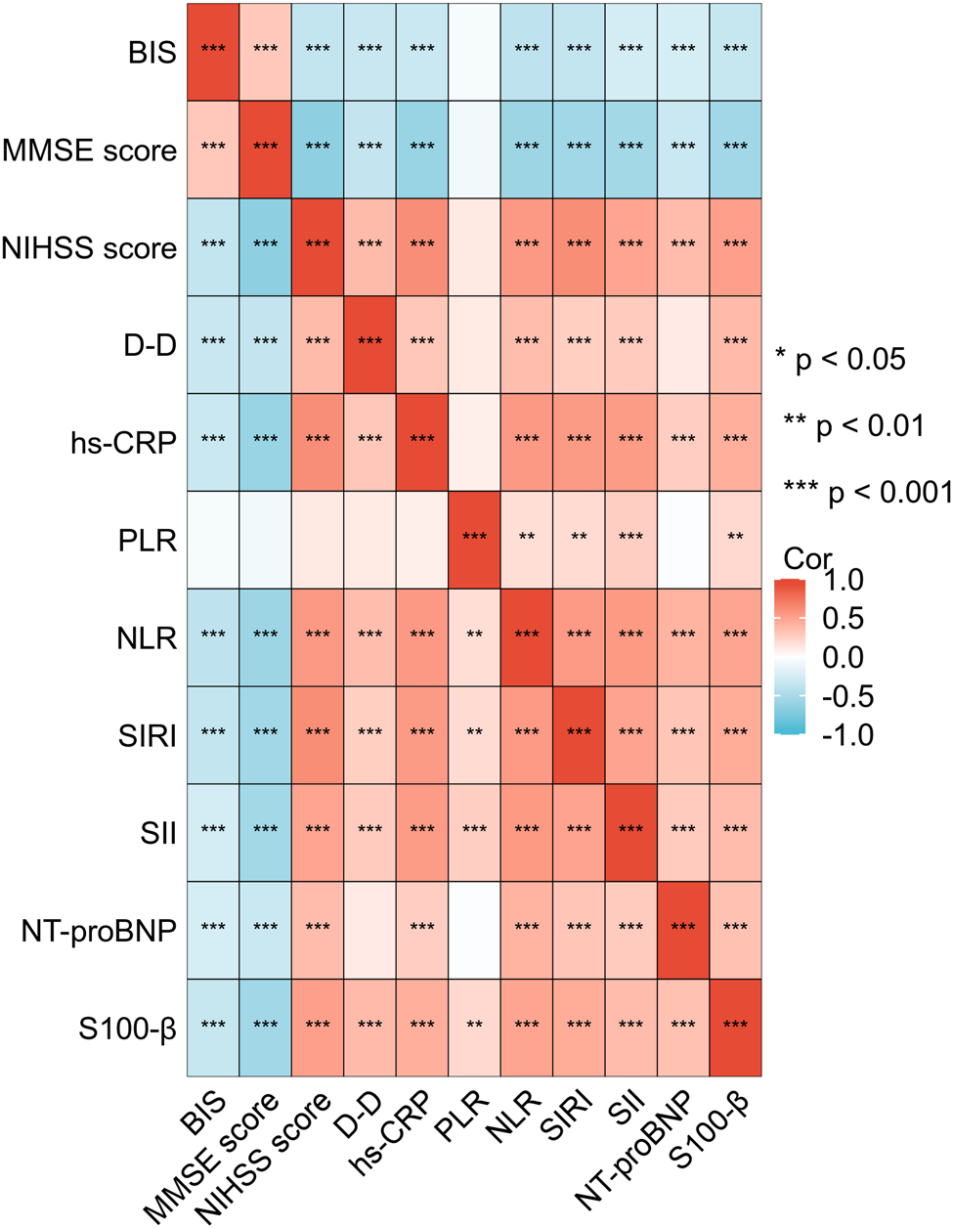
Correlation of BIS with clinical parameters in AIS patients. A total of 227 patients diagnosed with AIS were included, comprising individuals with high BIS (*n* = 163) or low BIS (*n* = 64) to analyze the correlation of BIS with clinical parameters, including NIHSS score, d-D, hs-CRP, NLR, SIRI, SII, NT-proBNP, and S100-β, in AIS patients with Spearman correlation.

A total of 227 patients diagnosed with AIS were included, comprising individuals with high BIS (*n* = 163) or low BIS (*n* = 64) to analyze the correlation of BIS with clinical parameters in AIS patients. Baseline data analysis revealed that several key indicators, including NIHSS score, d-D, hs-CRP, NLR, SIRI, SII, NT-proBNP, and S100-β, were significantly elevated in the low BIS group compared to the high BIS group (Table 4). However, MMSE score was significantly lower in the low BIS group compared to the high BIS group (Table 4). Then we performed spearman correlation analysis and found that BIS score was positively correlated with MMSE score (*r* = 0.300; *P* < 0.001). It was negatively correlated with NIHSS score (*r* = −0.364; *P* < 0.001), d-D (*r* = −0.331; *P* < 0.001), hs-CRP (*r* = −0.322; *P* < 0.001), NLR (*r* = −0.381; *P* < 0.001), SIRI (*r* = −0.359; *P* < 0.001), SII (*r* = −0.261; *P* < 0.001), NT-proBNP (*r* = −0.251; *P* < 0.001), and S100-β (*r* = −0.342; *P* < 0.001) (Figure 4).

**Table 4:** Baseline characteristics of AIS patients with high BIS (50–60) or low BIS (30–49)

Characteristics	High (*n* = 163)	Low (*n* = 64)	*P* value	Statistical method
MMSE score, median (IQR)	27.5 (27, 28.6)	21.4 (18.6, 24.9)	0.000	Wilcoxon
Age (years), median (IQR)	64 (58, 70)	63 (56, 72)	0.708	Wilcoxon
Gender, *n* (%)			0.541	Chisq test
Male	113 (49.8%)	47 (20.7%)		
Female	50 (22.0%)	17 (7.5%)		
Time of operation (min), median (IQR)	85 (72.0, 102.0)	90 (74.5, 105.0)	0.294	Wilcoxon
TOAST classification, *n* (%)			0.059	Chisq test
Cardioembolism	38 (16.7%)	14 (6.2%)		
Large-artery atherosclerosis	45 (19.8%)	24 (10.6%)		
Small vessel occlusion	67 (29.5%)	16 (7.0%)		
Others	13 (5.7%)	10 (4.4%)		
TICI, *n* (%)			0.621	Chisq test
2b-3	144 (63.4%)	58 (25.6%)		
0-2a	19 (8.4%)	6 (2.6%)		
Pneumonia, *n* (%)			0.072	Chisq test
No	99 (43.6%)	47 (20.7%)		
Yes	64 (28.2%)	17 (7.5%)		
Level of education, *n* (%)			0.366	Chisq test
Junior middle school or lower	99 (43.6%)	43 (18.9%)		
High school or higher	64 (28.2%)	21 (9.3%)		
LOS (days), median (IQR)	10 (8, 12)	10 (8, 11)	0.846	Wilcoxon
BMI (kg/m^2^), median (IQR)	24.2 (22.1, 26.6)	25.3 (23.5, 26.6)	0.419	Wilcoxon
Hypertension history, *n* (%)			0.693	Chisq test
No	105 (46.3%)	43 (18.9%)		
Yes	58 (25.6%)	21 (9.3%)		
SBP (mmHg), median (IQR)	150.0 (136.5, 161.0)	143.5 (132.0, 155.0)	0.129	Wilcoxon
DBP (mmHg), median (IQR)	87 (79, 95)	93 (83, 99)	0.000	Wilcoxon
Atrial fibrillation, *n* (%)			0.971	Chisq test
Yes	59 (26.0%)	23 (10.1%)		
No	104 (45.8%)	41 (18.1%)		
Diabetes history, *n* (%)			0.687	Chisq test
Yes	59 (26%)	25 (11%)		
No	104 (45.8%)	39 (17.2%)		
Stroke history, *n* (%)			0.754	Chisq test
No	108 (47.6%)	41 (18.1%)		
Yes	55 (24.2%)	23 (10.1%)		
NIHSS score, median (IQR)	4 (3, 5)	7 (5, 8)	0.000	Wilcoxon
Hemoglobin (g/dL), median (IQR)	11.1 (10.8, 11.8)	11 (10.8, 11.3)	0.408	Wilcoxon
Cre (mg/dL), median (IQR)	1.2 (0.9, 1.5)	1.2 (1, 1.5)	0.115	Wilcoxon
Hcy (μmol/L), median (IQR)	16.9 (14.6, 21.1)	16.8 (14.5, 20.9)	0.727	Wilcoxon
UA (μmol/L), median (IQR)	278.1 (176.4, 399.2)	241.7 (174.3, 410.2)	0.798	Wilcoxon
d-D (mg/L), median (IQR)	1.1 (0.8, 1.6)	1.6 (1.4, 1.8)	0.000	Wilcoxon
hs-CRP (mg/L), median (IQR)	4.9 (3.9, 6.9)	8.6 (5.3, 8.9)	0.000	Wilcoxon
PLR, median (IQR)	131.4 (114.7, 140.9)	133.7 (119.3, 143.5)	0.164	Wilcoxon
NLR, median (IQR)	2.8 (2.4, 3.3)	4.1 (3.45, 4.2)	0.000	Wilcoxon
SIRI, median (IQR)	1.6 (1.3, 2.7)	3.3 (3, 3.8)	0.000	Wilcoxon
SII, median (IQR)	486.3 (414.3, 603.2)	604.8 (535.7, 765.5)	0.000	Wilcoxon
TG (mmol/L), median (IQR)	1.9 (1.8, 2.1)	2 (1.9, 2.1)	0.056	Wilcoxon
TC (mmol/L), median (IQR)	4.5 (4, 5.15)	4 (3.8, 4.4)	0.000	Wilcoxon
LDL-C (mmol/L), median (IQR)	3.1 (2.7, 3.4)	3.05 (2.9, 3.3)	0.924	Wilcoxon
HDL-C (mmol/L), median (IQR)	1.2 (1.0, 1.4)	1.2 (1, 1.5)	0.235	Wilcoxon
NT-proBNP (ng/L), median (IQR)	421.3 (368.2, 490.4)	562.2 (445.5, 631.9)	0.000	Wilcoxon
S100-β (ng/L), median (IQR)	144.4 (126.5, 173.7)	244.95 (214.1, 272.8)	0.000	Wilcoxon

BIS, bispectral index; MMSE, mini mental status examination; IQR, interquartile range; TOAST, Trial of ORG 10172 in acute stroke treatment; LOS, length of stay; BMI, body mass index; TICI, thrombolysis in cerebral infarction; SBP, systolic blood pressure; DBP, diastolic blood pressure; NIHSS, National Institute of Health Stroke Scale; Cre, creatinine; Hcy, homocysteine; UA, uric acid; d-D, d-dimer; hs-CRP, high-sensitivity C reactive protein; PLR, platelet lymphocyte ratio; NLR, neutrophil lymphocyte ratio; SIRI, systemic inflammatory response index; SII, systemic immune inflammatory index; TG, triglyceride; TC, total cholesterol; LDL-C, low density lipoprotein cholesterol; HDL-C, high-density lipoprotein cholesterol; NT-proBNP, N-terminal pro-brain natriuretic peptide; S100-β, soluble protein-100 β.

## 4 Discussion

POCD is a prevalent complication among patients with AIS, with its incidence being associated with cerebrovascular lesions and strongly correlated with advancing age. Elderly individuals who have suffered a stroke are particularly vulnerable to developing POCD [26]. Following AIS in the elderly population, localized tissue ischemia may directly precipitate oxidative stress and inflammation, ultimately leading to brain injury [27]. Brain tissue is highly susceptible to hypoxia, and delayed irreversible nerve damage can occur even with timely thrombolysis and thrombectomy [27]. Previous studies have indicated that CRP, NLR, reduced HDL-C, BMI, NIHSS score, and SBP are risk factors for perioperative POCD [3,28–30]. Li et al. identified elderly age, the presence of carotid artery plaques, a high NIHSS score, multiple infarct lesions, and specific infarct types as significant risk factors for cognitive dysfunction in patients recovering from cerebral infarction [31]. Tang and colleagues [32] employed gender, age, baseline NIHSS score, hyperhomocysteinemia, and multiple lesions in the development of a nomogram model for the prediction of acute cognitive impairment following stroke, achieving an AUC value of 0.859. In light of the significance of the inflammatory response and brain injury in contributing to cognitive dysfunction, we utilized multivariate regression analysis to identify two inflammatory indicators (hs-CRP and SII) and three brain injury indicators (NIHSS score, NT-proBNP, and S100-β), which were used to construct a nomogram model. Evaluation through calibration curves, DCA, and PR curves demonstrated the satisfactory predictive performance of the nomogram model. Our results reaffirm the role of elevated inflammation and increased brain damage in exacerbating POCD in patients with AIS.

Numerous studies have established a correlation between inflammation and cognitive impairment, with inflammation identified as a potential mechanism contributing to POCD [33,34]. Impairment of the blood–brain barrier further intensifies central inflammation, facilitating the infiltration of peripheral immune cells into the brain and fostering inflammation [33–35]. Peripheral inflammation is recognized as a primary factor in inducing neuroinflammation within the brain, as pro-inflammatory cytokines have been observed to breach the blood–brain barrier and incite central inflammatory responses [35–37]. Guo et al. [38] indicated a significant negative relationship between systemic inflammation markers, including SIRI, SII, pan-immune-inflammation value, and cognitive functioning, implying that targeting inflammation may be a viable strategy for improving cognitive well-being and reducing cognitive decline associated with aging. In comparison to patients without POCD, those with POCD exhibited significantly higher SII and SIRI values. Both SII and SIRI were found to have negative correlations with Montreal cognitive assessment scores. Furthermore, multivariable logistic regression analysis revealed that SII was independently associated with POCD, while SIRI was not significantly associated [39]. Consistent of these findings [38,39], this study revealed a significant upregulation of SII and hs-CRP in patients with POCD compared to those without POCD in AIS patients. Furthermore, both SII and hs-CRP were identified as independent risk factors for the development of POCD in AIS patients.

The present findings suggest that there is no statistically significant disparity in the prevalence of cognitive impairment among elderly individuals following general anesthesia compared to regional anesthesia [19]. The outcomes of both anesthesia modalities remained comparable for a duration of up to 7 days post-surgery, implying that the choice of anesthesia may be contingent upon the unique characteristics of each patient rather than anticipated variations in clinical consequences [19,20,40]. While previous research has indicated that the depth of anesthesia may not have a direct impact on cognitive function, this study categorizes AIS patients undergoing general anesthesia based on BIS score with high score (50–60) or low score (30–49). Our findings demonstrate a significant difference in MMSE scores between the low BIS and high BIS groups, implying that depth of anesthesia could potentially contribute to cognitive decline in patients receiving general anesthesia. Our findings indicate a significant negative correlation between BIS score and various clinical measures, such as NIHSS score, d-D, hs-CRP, PLR, NLR, SIRI, SII, NT-proBNP, and S100-β. Increased depth of anesthesia may be linked to brain injury and inflammatory response, with five of these parameters identified as independent risk factors for the development of POCD. Overall, the depth of anesthesia may be a factor in the occurrence of POCD in AIS patients undergoing general anesthesia. However, Spearman’s correlation coefficient indicates that the correlation between depth of anesthesia and cognitive impairment is not a strong correlation. Consequently, further investigation in a larger sample is required to validate this relationship.

Our research successfully developed a novel nomogram model for predicting POCD in patients with AIS, demonstrating a strong level of accuracy. Additionally, our findings suggest a potential association between anesthesia depth, cognitive impairment, and inflammatory response. However, it is important to acknowledge the limitations of this study. Initially, the study did not include an analysis of the comparative impacts of varying anesthesia types on cognitive function. Additionally, the examination did not address the potential effects of narcotic drug utilization on cognitive function. Furthermore, while a correlation was identified between the anesthesia depth and cognitive impairment and inflammatory response, the strength of this association was deemed insufficient and necessitated validation through a more extensive sample size. Finally, this study did not include parameters related to perioperative complications. Future research will aim to investigate the impact of perioperative complications on POCD.

## 5 Conclusion

In summary, our findings indicate that the composite predictive model incorporating NIHSS score, hs-CRP, SII, NT-proBNP, and S100-β demonstrates efficacy in predicting POCD following AIS. Additionally, our results suggest a potential association between depth of anesthesia, cognitive impairment, and inflammatory response in AIS patients.
